# 

**Published:** 1890-08

**Authors:** 


					﻿Entered at the Post Office at Austin. Toras, as Second Class Man Matter.
Vol. VI
AUGUST. 1890
No. 2.
DANIEL'S
MEDECAL
JOURNAL
F. E. DANIEL., M.D., Editor and Publisher, AUSTIN, TEXAS.
Eugene Von Boeckmann. Steam Book and Job Printer. Aurtin, Texas.
				

